# The clinicopathologic features and prognosis of esophageal neuroendocrine carcinomas: a single-center study of 53 resection cases

**DOI:** 10.1186/s12885-019-6420-8

**Published:** 2019-12-18

**Authors:** Lei Ye, Heng Lu, Lin Wu, Lei Zhang, Hui Shi, Hui Min Wu, Pin Tu, De Min Li, Fang Yu Wang

**Affiliations:** 1 Department of Gastroenterology and Hepatology, Jinling Hospital, Medical School of Nanjing University, 305 Zhongshan East Road, Nanjing, Jiangsu Province, 210002 China; 2 Department of cardiothoracic surgery, Jinling Hospital, Medical School of Nanjing University, 305 Zhongshan East Road, Nanjing, Jiangsu Province, 210002 China; 3 Department of Pathology, Jinling Hospital, Medical School of Nanjing University, 305 Zhongshan East Road, Nanjing, Jiangsu Province, 210002 China

**Keywords:** Esophageal neuroendocrine carcinomas, Clinicopathologiccharacteristics, Prognosis, Retrospective study

## Abstract

**Background:**

Esophageal neuroendocrine carcinomas (NECs) are exceedingly rare and poorly understood. The aims of the retrospective study were to delineate the clinicopathologic features and prognosis of patients with the disease.

**Methods:**

We performed a retrospective study containing 53 patients of esophageal NECs in our center from 2002 through 2018. Patients were assigned to the pure esophageal NECs group and the esophageal NECs mixed with squamous carcinoma and/or esophageal adenocarcinoma (MiNECs) group. Demographic, clinical, pathologic and prognostic factors were recorded and analyzed.

**Results:**

Of the 53 patients, elderly male patients were predominant. Dysphagia was the most common symptom (45/53, 84.9%). Most tumors were centered in the middle esophagus (36/53,67.9%).Ulcerated appearance was frequently seen in the pure NECs (56.8%), and the tumors in the MiNECs group mostly represented elevated types (57.9%). Synaptophysin (38/45, 84.4%), chromogranin A (21/38, 55.3%) and CD56(23/27, 85.2%) have been proven to be positive markers for NECs. Most patients (46/53, 86.8%) received surgery combined with chemotherapy. Though the pathologic stages were alike (*P* = 0.129), the median survival time was 3.53 years for the pure NECs group and 7 years for the MiNECs group. In multivariate analysis, pathologic stage (RR = 1.938, *P* = 0.045) and age (RR = 2.410, *P* = 0.028) were independent prognostic factors for patients with MiNECs. The prognosis of patients with pure NECs was independent from any factors.

**Conclusions:**

Careful endoscopic examination could help distinguish pure NECs from MiNECs. NECs were aggressive, but a relative better prognosis for patients with MiNECs. Surgery should be performed if applicable, and chemotherapy might be helpful.

## Background

Gastrointestinal neuroendocrine carcinomas (GINECs), originating from stem cells and characterized by producing polypeptide hormones, have witnessed an increase in incidence worldwide due to increased physician awareness and improved diagnostic modalities [1, 2]. However, NECs in the esophagus are extremely rare, and data on the clinical features, pathological descriptions and prognosis is limited. Most published literature is case reports, and so far, the largest study coming from West China Hospital has reported a total of 49 cases of esophageal NECs [3]. Because of the paucity of large surveys regarding experience with esophageal NECs, the biological features and optimum therapy far from being established up. Therefore, studies about the disease with relatively large sample size are badly needed. Here, we reported our single center experience of 53 cases of esophageal NECs diagnosed between 2002 and 2018. In this study, we described the clinical and pathological features, immunohistochemical findings, endoscopic characteristics and prognosis of primary esophageal NECs. Co-existence of squamous cell carcinoma and/or adenocarcinoma is often observed [4]. To better understand the behavior of the NECs, we categorized patients into groups with and without synchronous esophageal tumors. To our knowledge, this is the largest clinic-pathological study investigating the entity to date.

## Methods

### Patients

A retrospective analysis of patients diagnosed with primary esophageal NECs from 2002 to 2018 in our center was done. All patients have been treated by radical open thoracic esophagectomy or palliative surgery. Inclusion criteria were cases with final pathologic diagnoses of esophageal NECs, regardless of pure NECs or esophageal squamous carcinoma with neuroendocrine differentiation or esophageal adenocarcinoma with neuroendocrine differentiation. Exclusion criteria were: 1) clinical data were not available even though the pathologic diagnosis was an esophageal NEC; 2) the patient had a history of neuroendocrine carcinoma in other organs; 3) the final pathological diagnosis was made on the biopsy sample; 4) the tumors located in the gastroesophageal junction (GEJ) were carefully evaluated, and only carcinomas in which the epicenter was present within 2 cm to the GEJ were included [5]. The study protocol was approved by the Medical Ethics Committee of the Jinling Hospital.

### Clinical data collection

Patient age, gender and presenting symptoms were extracted from the electronic medical records. The esophageal tumor locations were divided based on endoscopic findings into upper (15 to 24 cm from the incisor teeth), middle (24 to 32 cm from the incisor teeth), and lower (32 to 40 cm from the incisor teeth) [6]. Tumor gross appearances were extracted from the pathologic reports and categorized into 3 major groups: polypoid or nodular elevated types, ulcerated and flat.

### Pathologic staging and classification

Due to a lack of standardized staging guidelines of the cancer, we chose to stage this cancer with the rules for esophageal squamous carcinoma defined by the American Joint Cancer Committee (AJCC) in the 8th edition of the cancer staging manual [5]. Grading of the NECs was done in accordance with the 2017 World Health Organization (WHO) classification for neuroendocrine tumors [7]. Briefly, it was proposed to adopt the term “neuroendocrine neoplasm (NEN)” as a term encompassing all tumor classes. The well-differentiated family was designated “neuroendocrine tumor (NET)”, and the poorly differentiated family “neuroendocrine carcinoma (NEC)”. Mixed neuroendocrine-non-neuroendocrine neoplasm in the pancreas was designated as MiNEN, and mixed neuroendocrine-non-neuroendocrine carcinoma could be named as MiNEC. Mixed adenoneuroendocrine carcinoma in the tubular gastrointestinal tract was named as MANEC. These comlex neoplasms are common, but they were not included in the present classification framework.

For NENs, tumors were categorized into three levels (G1, G2 and G3) based on mitotic or Ki-67 labeling index. Though necrosis was recognized as a potential adverse prognostic factor, it was not included in the grading parameters. Mitosis was graded into G1(< 2/10 higer power fields, HPF), G2(2–20/10 HPF) or G3(> 20/10 HPF). The Ki-67-labeling index was graded into G1(≤2%), G2(3–20%) or G3(> 20%). Tissue blocks were cut into 4-μm thick sections. The average number of tumor-containing slides reviewed was 4(range 2 to 8) per case. Since all cases of the study were G3 NECs, esophageal squamous carcinoma with neuroendocrine differentiation or esophageal adenocarcinoma with neuroendocrine differentiation were categorized into MiNECs. Results of immunohistochemical staining were extracted from the pathologic reports and presented as either positive or negative.

### Survival outcomes

Patients’ prognosis information after resection was acquired through telephone with the patient or family members. For patients who have changed their telephone numbers, we contacted with the Area Population Bureau to obtain their survival information. However, there were two patients whose recorded information has been deleted and five patients who died but no definite death time was recorded. Thus, these seven patients were classified into follow-up loss, and they were excluded in the survival analysis.

### Statistical analysis

Statistical analyses were performed using the Statistical Package for Social Sciences (SPSS) version 18 software (SPSS Inc., Chicago, IL, USA). Continuous variables with parametric distribution are presented as the mean ± standard deviation, and median and interquartile range (IQR) for non-parametric distribution. Student t test was used to analyze the differences between the two independent groups. For continuous variables with a non-parametric distribution, the differences between groups were compared using the Mann-Whitney U test, and categorical variables were compared using the chi-squared test. Life-table analysis was used to calculate the median survival time for patients. Survival curves were displayed by Kaplan-Meier analysis with the log rank test. In patients who were alive at the last follow-up, survival rates were censored. A Cox proportional hazards regression (using the “Forward: LR” methods) was used to examine the independent prognostic factors by calculating the hazard ratio (HR) and 95% confidence interval (CI) in multivariate analysis. A *p*-value less than 0.05 was considered statistically significant.

## Results

### Clinical characteristics

The clinical characteristics of the 53 primary esophageal NECs were shown in Table 1. Among these patients, there were 36 patients who were pathologically diagnosed with pure NECs. To observe whether there exhibited some differences between patients with pure NECs or patients with MiNECs, we summarized them separately. The differences between the two groups were not statistically significant in all clinical parameters analyzed. The mean age of patients in the pure NECs group was 59.92 ± 7.96 (54.25–65.00) years old, and that of the MEET group was 62.76 ± 9.41(55.00–69.50) years old. Both groups were male-dominant, with a proportion 63.9 and 76.5% separately. Forty-five out of 53 patients presented to our institution with dysphagia. Weight loss was the second common symptom. Other presenting complaints like chest pain or hot flushes seemed to be more reported in the pure NECs group than those in the MiNECs groups, but there was no significant difference. Abdominal discomfort at diagnosis was recorded in 6 pure NECs patients (16.7%) and 4 MiNECs patients (23.5%). Melena was documented in only one patient. There was no patient exhibiting typical carcinoid symptom in our study.

**Table 1:** Demographic characteristics and clinical symptoms of esophageal neuroendocrine carcinomas

	Pure NECs^+^ (%)	MiNECs^+^ (%)	*P*
Mean Age(y^+^)	59.92 ± 7.96	62.76 ± 9.41	0.257
Sex (Male)	23(63.9)	12(76.5)	0.360
Chief complaints			0.350
Asymptomatic	0 (0.0)	1(5.9)	
Dysphagia	32(88.9)	13(76.5)	
Abdominal discomfort	6(16.7)	4(23.5)	
Weight loss	9(25.0)	4(23.5)	
Melena	0 (0.0)	1(5.9)	
Hot flushed	2(5.6)	0 (0.0)	
Chest pain	8(22.2)	3(17.6)	

^+^NECs: neuroendocrine carcinomas; MiNECs: Mixed neuroendocrine-nonneuroendocrine carcinomas; y: years

### Endoscopic findings

**Fig. 1:**
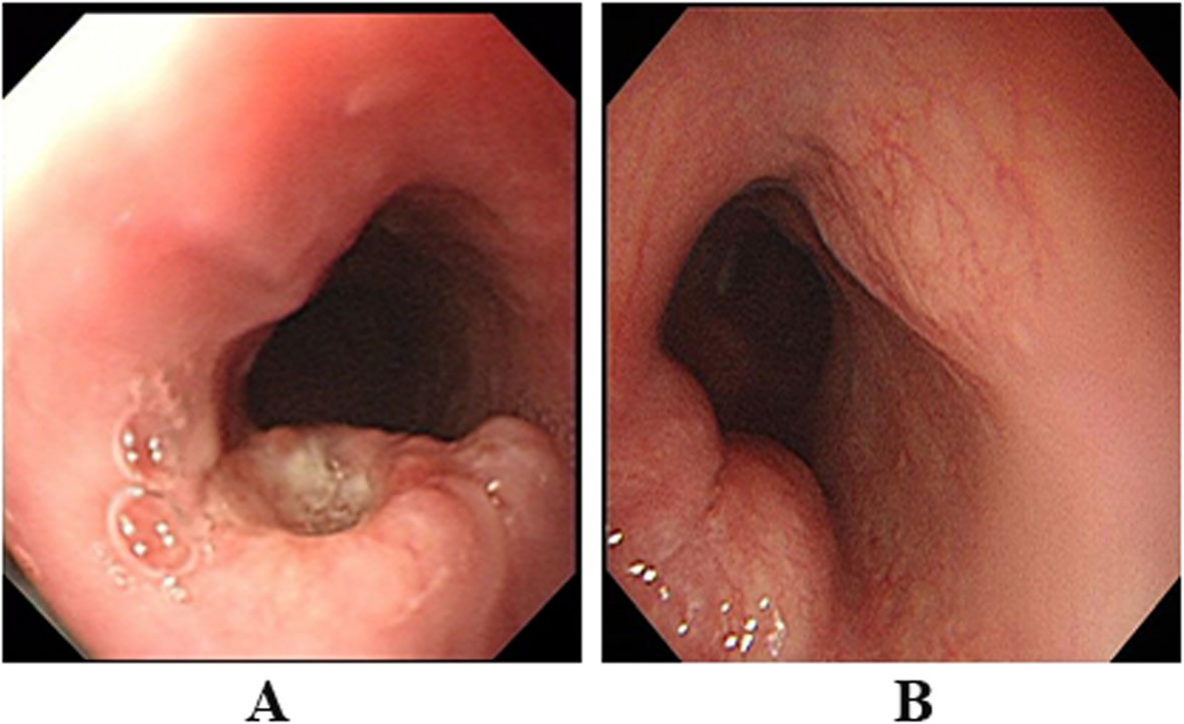
Typical endoscopic findings of esophageal NECs. (A) Elevated and depressed types for the pure NECs; (B) Nodular elevated types with glistening overlying surface for the MiNECs

The endoscopic findings of the 53 cases of primary esophageal NECs were summarized in Table 2. Majority were centered in the middle esophagus, with 72.2% of the pure NECs and 58.8% of the MiNECs. Tumors were mainly represented in a single lesion (35/36, 15/17, respectively). A total of 3 patients were found to have two lesions in the esophagus. The median size of tumors in the pure NECs was 3.0 cm (2.35–4.50), and 4.0 cm (3.0–4.75) for MiNECs. The gross appearance of the two groups had significant differences, with *p* value < 0.01. Ulcerated gross appearance was frequently seen in the pure NECs (56.8%), and more specifically, some of the ulcerated tumors exhibited upon elevated lesions which could be described as elevated and depressed types (Fig. 1a). In contrast, the tumors in the MiNECs group mostly represented polypoid or nodular elevated types (57.9%) with glistening overlying surface (Fig. 1b). Flat tumors were seen in 2 cases of the pure NECs group and 6 cases of the MiNECs group respectively.

**Table 2:** Macroscopic characteristics of esophageal neuroendocrine carcinomas

	Pure NECs^+^(%)	MiNECs^+^(%)	*P*
Tumor location			0.049
Upper (15-24 cm)	4(11.1)	0(0.0)	
Middle (24-32 cm)	26(72.2)	10(58.8)	
Lower (32-40 cm)	6(16.7)	7(41.2)	
Tumor numbers			0.493
Single	35(97.2)	15(88.2)	
Two	1(2.8)	2(11.8)	
Three or more	0(0.0)	0(0.0)	
Tumor size (cm)			0.275
Median (range)	3.0(2.35–4.50)	4.0(3.0–4.75)	
Gross appearance			0.001
Polypoid nodular elevated types	14(37.8)	11(57.9)	
Flat	2(5.4)	6(31.6)	
Ulcerative	21(56.8)	2(10.5)	

^+^NECs: neuroendocrine carcinomas; MiNECs: mixed neuroendocrine-nonneuroendocrine carcinomas

### Pathologic staging

In resection specimens, each group had one patient lacking detailed pathologic stage report, thus the 8th edition of the cancer staging manual could not be applied. And the top three stages of both groups were IIA (15/35, 42.9%; 7/16, 43.8%), IIIB (7/35, 20.0%; 5/16, 31.25%) and IVB (3/35, 8.6%; 2/16, 12.5%). Likewise, there was no significant differences between the pure NECs group and the MiNECs group in the lymph node metastasis and distant metastasis (Table 3). Specifically, the MiNECs group seemed to be more aggressive compared with the pure NECs. As it shown in Table 3, that tumors invaded the outer membrane of esophagus happened in 11 out of 16 patients from the MiNECs group. By contrast, tumors invading the esophageal muscular layer were predominant in the pure NECs group.

**Table 3:** Pathologic stage of esophageal neuroendocrine carcinomas

	Pure NECs^+^(%)	MiNECs^+^(%)	*P*
Total number	35(97.2)	16(94.1)	
pT			0.021
1a	0(0.0)	0(0.0)	
1b	8(22.9)	1(6.25)	
2	15(42.9)	4(25.0)	
3	12(34.3)	11(68.8)	
4	0(0.0)	0(0.0)	
pN			0.194
0	23(65.7)	7(43.8)	
1	6(17.1)	5(31.3)	
2	5(14.3)	4(25.0)	
3	1(2.9)	0(0.0)	
pM			1.000
0	32(91.4)	14(87.5)	
1	3(8.6)	2(12.5)	
Summary stage			0.129
IA	0(0.0)	0(0.0)	
IB	7(20.0)	0(0.0)	
IIA	15(42.9)	7(43.8)	
IIB	0(0.0)	1(6.25)	
IIIA	2(5.7)	1(6.25)	
IIIB	7(20.0)	5(31.25)	
IVA	1(2.9)	0(0.0)	
IVB	3(8.6)	2(12.5)	

^+^NECs: neuroendocrine carcinomas; MiNECs:mixed neuroendocrine-nonneuroendocrine carcinomas

All tumors were staged according to the rules for esophageal squamous carcinoma (AJCC8)

### Immunohistochemistry

**Fig. 2:**
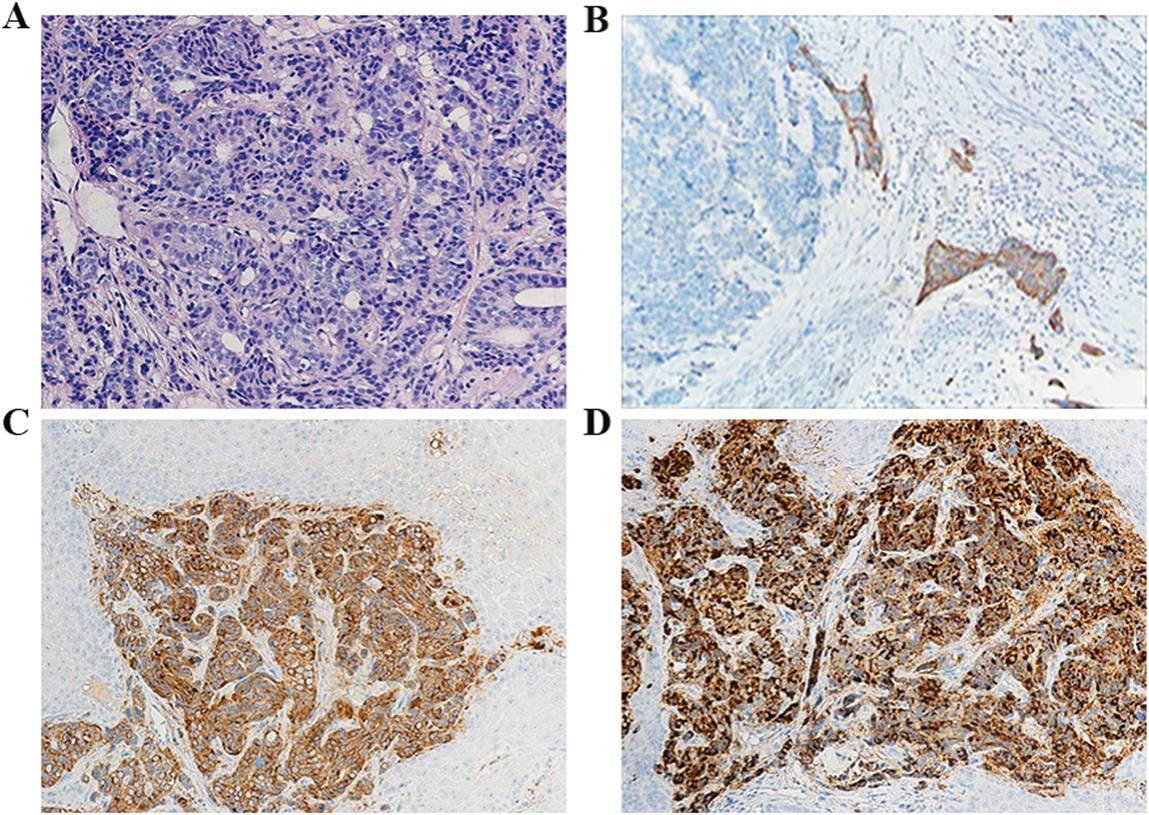
Representative esophageal NECs with minor components of squamous cell carcinoma. (A) Neoplastic cells exhibited cribriform growth pattern and small oat-like features, and tumors stroma was rich in venules (hematoxylin-eosin stain); (B) The component of squamous cell carcinoma was strongly immunoreactive to the CK5/6 antibody;(C) Neoplastic cells exhibited immunoreactivity to synaptophysin; (D) Robust, diffuse immunoreactivity to chromogranin A was observed (Immunostains in B-D)

A definite pathologic diagnosis of NET requires demonstration of essential neuroendocrine features in immunohistochemistry. Table 4 exhibited the markers that our center adopted in the pathologic reports. The results indicated that neoplastic cells in the pure NECs displayed strong immunoreactivity to both synaptophysin (86.7%) and chromogranin A (64.0%). Robust immunoreactivity was also observed to CD56 (87.5%) and CKpan (81.3%). Immunostains for p63, CK5/6,CK8/18 and CK7 were also positive to various degrees. Negative immunoreactivity to p63 and CK5/6 was characteristic. None of the only eight cases tested was found positive for p40 in NECs. However, two out of three cases showed robust p40 immunoreactivity in MiNECs, which might indicate that p40 could be the potential negative marker for NECs. Histopathological figures about representative cases were shown in Fig. 2.

**Table 4:** Positive immunohistochemical profile of esophageal neuroendocrine carcinomas

Antibody	Pure NECs^+^(%)	MiNECs^+^(%)	P
Syn^+^	26/30(86.7)	12/15(80.0)	0.884
CgA^+^	16/25(64.0)	5/13(38.5)	0.178
CD56	14/16(87.5)	9/11(81.8)	1.000
P63	2/26(3.8)	6/8(75.0)	0.001
CK5/6	1/18(5.6)	6/10(60.0)	0.003
CK8/18	4/5(80)	3/4(75)	0.858
CKpan	13/16(81.3)	3/4(75)	0.784
P40	0/8(0)	2/3(66.7)	0.010
CK7	2/4(50.0)	4/4(100)	0.063

^+^NECs: neuroendocrine carcinomas; MiNECs: mixed neuroendocrine-nonneuroendocrine carcinomas; Syn: synaptophysin; CgA: chromogranin A

### Patient survival

**Fig. 3:**
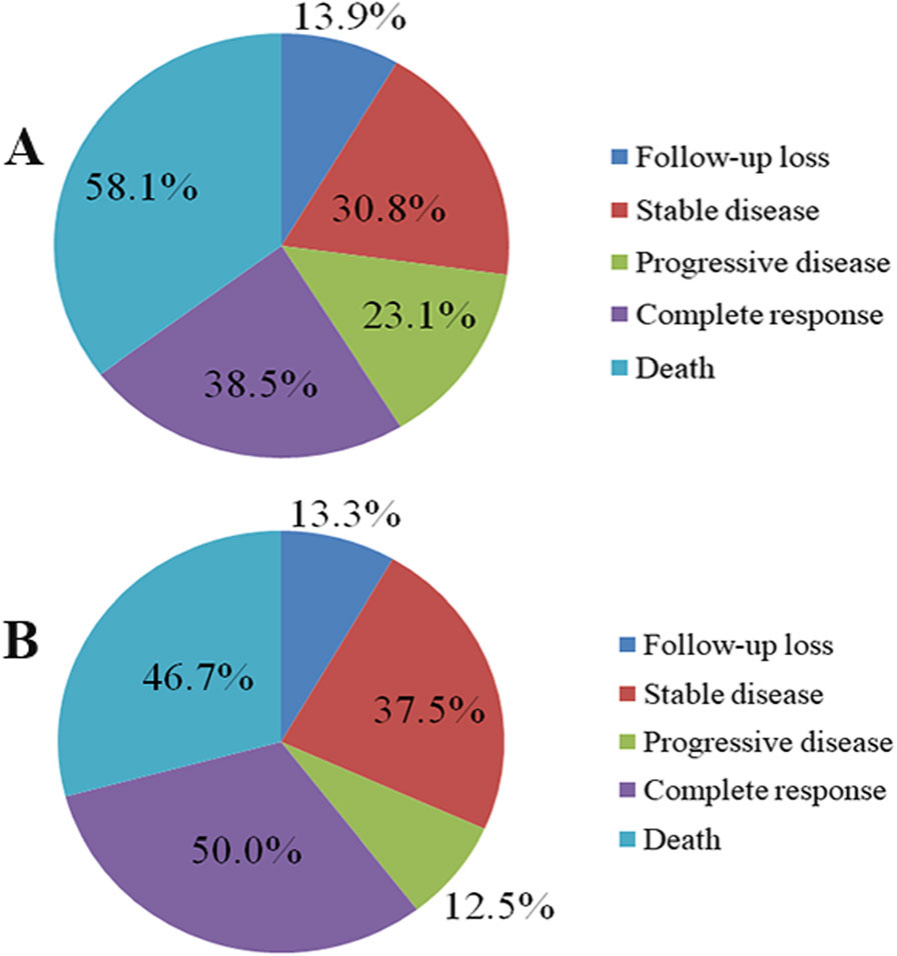
Treatment response of esophageal NECs. (A) Treatment response of the pure NECs; (B) Treatment response of the MiNECs

In the study, there were a total of seven patients who were lost to contact, five in the pure NECs group and two in the MiNECs group. Eighteen out of thirty-one patients (18/31, 58.06%) had died, and in the remaining alive patients, five patients (5/13, 38.46%) achieved complete response, four (4/13, 30.77%)were in stable disease and the last three patients (3/13, 23.08%) unfortunately developed multiple metastases (Fig. 3a). In contrast, seven patients (7/15, 46.67%) had died in the MiNECs group. Four out of eight (4/8, 50.00%) patients alive achieved complete response, three patients (3/8, 37.50%) were in stable disease and one (1/8, 12.50%) developed multiple metastases (Fig. 3b).

**Fig. 4:**
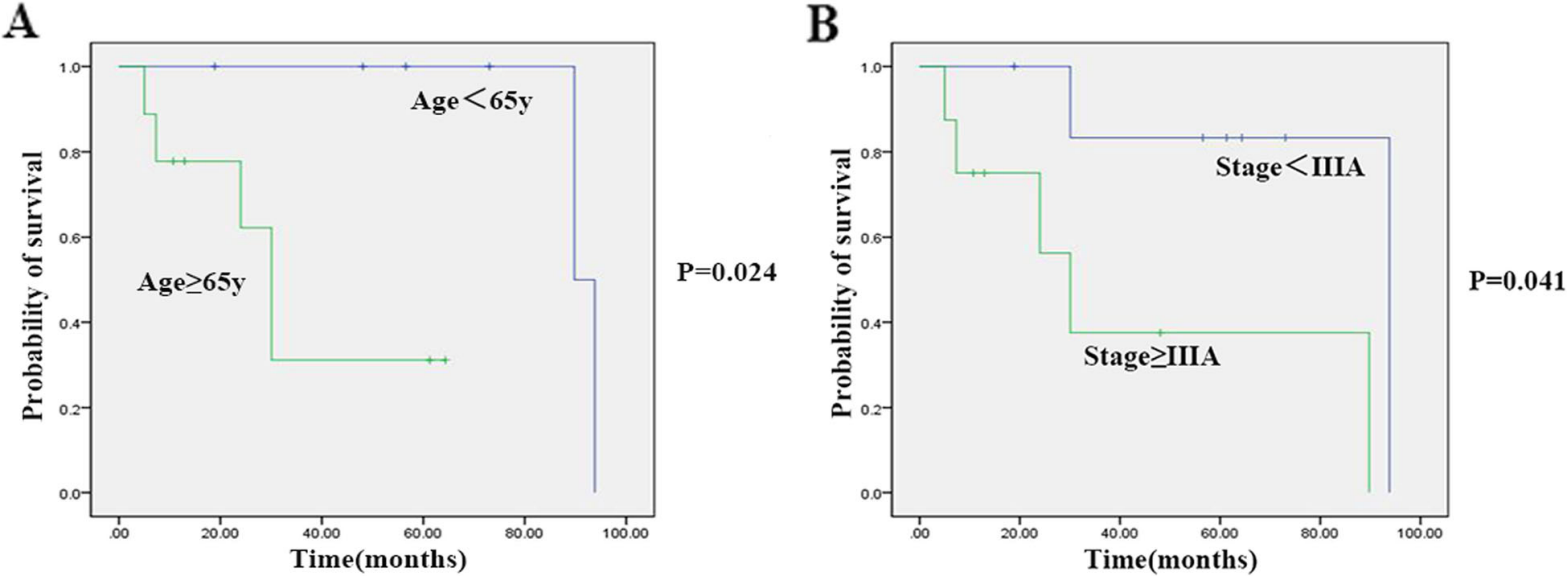
Kaplan-Meier survival curve for patients with esophageal MiNECs. (A) Patients older than 65 years old appeared to have significantly worse survival compared with those younger than 65 years old; (B) Patients with pathologic stage higher than IIIA appeared to have significantly worse survival compared with those with pathologic stage lower than IIIA

Using life-table analysis, the median survival time was 3.53 years for the pure NECs group and 7 years for the MiNECs group. The five-year survival rates for the two groups were 46 and 60%, respectively. The Cox proportional hazards regression showed there existed no independent prognostic factors for the pure NECs group, which meant that once the patient was diagnosed with NEC, then the five-year survival rate was 46% for him regardless of age, sex, tumor sizes or pathological stage. On the other hand, the possible independent prognostic factors for patients with MiNECs were pathologic stage (RR = 1.938, *P* = 0.045) and age (RR = 2.410, *P* = 0.028) (Table 5). Moreover, Kaplan-Meier survival curves based on age and pathologic stage were calculated (Fig. 4a, b). The results demonstrated that the median survival time for patients older than 65 years old was 30 months, which was significantly shorter than patients younger than 65 years old (Median survival time 90 months, *P* = 0.024,< 0.05). Similarly, stage IIIA could be another prognostic factor (*P* = 0.041,< 0.05).

**Table 5:** Cox regression analysis for patients with MiNECs^+^

	RR^+^	95%CI^+^	*P*
Stage	1.938	1.016–3.694	0.045
Age	2.410	1.097–5.292	0.028

^+^ MiNECs: mixed neuroendocrine-nonneuroendocrine carcinomas; RR: risk ratio; CI: confidence interval

## Discussion

The first description of the esophageal NECs was reported by Mckeown in 1952 [8]. Since then, the publicity of the disease was widened especially after the WHO definition. Though a bulk of literature has been published, most of them were case reports. And data summarizing the characteristics of the disease were still badly scarce. Compared to several published single or multicenter studies on the subject [3, 4, 9–12], our study is the largest and longest-running single center clinicopathologic study worldwide.

NECs contain neuroendocrine cells that secrete bioactive substances and the WHO definition for NET includes positive endocrine markers such as chromogranin A. In this study, we have analyzed 53 cases of NECs of the esophagus confirmed by the tissue pathology after surgery. Consistent with other studies [4, 13], our results showed that the widely accepted positive immunostaining should be synaptophysin, CD56 and chromogranin A. CK8/18 and CKpan could be candidate markers. Besides, p63, p40 and CK5/6 have been demonstrated to be potent markers of the squamous cell carcinoma or adenocarcinoma. P53 loss has been found nearly universal in poorly differentiated NECs [14], and it was a limitation that our center didn’t detect p53 in the surgical specimens. Interestingly, only one patient whose biopsy sample by endoscopy indicated neuroendocrine carcinoma, others reported esophageal squamous carcinoma or esophageal adenocarcinoma, which meant a high misdiagnosis from biopsy (data not shown).

Like other malignancies of the esophagus, the vulnerable patients were elderly males. Dysphagia was the most common symptom, followed by weight loss. Tumors were frequently found in the middle esophagus, and this could be the reason for the phenomenon of synchronous esophageal squamous carcinoma. It has been reported that esophageal squamous carcinomas were most frequently present in the middle esophagus [15, 16]. Protruding type with ulceration in the center was the obvious features of the pure NECs, which was different from the elevated type of the MiNECs. It could be attributed that the squamous component often overlies NECs [13].

Our study was a retrospective study analyzing all eligible patients from 2002 to 2018. The follow-up period was 16 years, and the unexpected thing was that some patients with pure NECs have lived longer than 5 years, since NECs are aggressive and the prognosis reported in literature is poor [4, 9–12, 17]. Specifically, there were several patients with localized pure NECs or NECs with small part of squamous cell carcinoma achieved complete response after resection. The longest survival time was more than 10 years. Several case reports also demonstrated that patients could achieve complete response after surgery despite the aggressive behavior of the disease [18–20]. The shared feature of these patients was that tumors were localized and could be completely removed.

The median survival year for patients with pure NECs was 3.53 years, and 7 years for patients with MiNECs, which was different from some studies. Kanakasetty et al. have summarized data in their center and concluded that the median survival time for pure NECs patients without metastasis was 18.25 months and only 6.5 months for patients with lymph node positive [9]. In the study by Egashira et al., the median survival time of patients with non-metastasis was 17 months and as short as 8.5 months for patients with tumors outside loco-regional boundaries [4]. Reports from western countries showed that the median survival for small cell carcinoma of the esophagus was 11 months [21]. However, the study by Huang et al. showed a better prognosis for patients after resection. The median survival time for high-grade neuroendocrine carcinomas was 28.5 months, which was approximate to ours. Lymphovascular and organ invasion were found to be of prognostic value [4, 9, 11, 13]. And Lee et al. reported that tumor size (more than 2.0 cm) showed prognostic significance [11]. Different from these results, we found that there were no prognostic factors for patients with pure NECs. Once the patient was definitely diagnosed, his median survival time was 3.53 years, and the 5-year survival rate was 46%, independent from any factor such as tumor size. For patients with MiNECs, age and pathologic stage were found to be significantly associated with prognosis. Patients older than 65 or pathologic stage higher than IIIA had worse prognosis. Overall, the 5-year survival rate for patients with MiNECs was 60%. The heterogeneity of the prognosis could be attributed to many factors, and the main one should be the small sample size of all of the studies. Others could be the quality of pathologic reports or the inclusion criteria of patients or treatment modality.

Due to lack of sufficient data about the clinicopathological characteristics of esophageal NECs, there has no established treatment yet. Surgery, chemotherapy and radiotherapy are recommended [22, 23]. In our center, for operable patients, surgery was the first choice. Even for inoperable NECs, palliative surgery was conducted. Adjuvant chemotherapy was always followed if the patient’s condition was allowed. In the study, except for seven patients who lost to follow-up, the remaining patients received chemotherapy after resection. There was one patient receiving radiotherapy. Although the co-existence of squamous cell carcinoma with NECs makes complete response by chemotherapy difficult, our study indicated that adjuvant chemotherapy for operable patients provided a better prognosis, which was also reported in other studies [1, 3, 24]. Nevertheless, detailed information about adjuvant therapy was omitted because most patients received chemotherapy in local hospitals. Cisplatin/etoposide and cisplatin/irinotecan were two major regimens for those patients in our center, which was also confirmed in other studies [25–27]. Furthermore, recent evidence from sequencing provides potential therapeutic strategies on the basis of genetics and epigenetics [14].

A major limitation of the work is that it was a retrospective study, and therefore, some data such as chemotherapy information were omitted and pathologic reports were not standardized. As a result, immunohistochemical profile of every case was not consistent. However, a prospective trial cannot be envisioned because of the rarity of the disease. The overall sample size is reasonably large for the rare disease. Besides, our retrospective study has a relatively low rate of loss to follow-up (13.2%) in the follow-up period of 16 years. Overall, the validity of our data is strengthened by the fact that the study included patients who were diagnosed by resection specimens rather than clinical characteristics or biopsy samples. Regardless, the findings of the study require larger studies to confirm and update.

## Conclusions

The study of 53 resected esophageal NECs providing detailed information on clinical, pathologic, immunohistochemical and prognostic features, is the largest study reported to date. Different from published studies, NECs were analyzed by divided into pure NECs and theMiNECs to better guide clinic practice. The results demonstrated that careful endoscopic examination could assist in assessing whether there exist synchronous esophageal tumors or not. Pure NECs tend to present ulcerated types, and MiNECs are polypoid or nodular. This is crucial, because MiNECshave a better prognosis than pure NECs. Age and pathologic stage are the only two significant prognostic parameters forMiNECs. Whenever feasible, surgery is the mainstay treatment. Complete resection conferred a significant survival advantage. Yet, whether chemotherapy helps in symptom palliation and improves survival should be illustrated in prospective or larger retrospective studies. Moreover, recent biological findings might provide potential therapeutic strategies targeting the molecular mechanisms. We are looking forward to witness a better identification and optimal therapeutic strategy for the rare entity.

## Data Availability

The datasets generated during the study are available from the corresponding author on reasonable request.

## Abbreviations

AJCC: American Joint Cancer Committee
CgA: Chromogranin A
CI: Confidence interval
IQR: Interquartile range
MiNECs: Mixed neuroendocrine-nonneuroendocrine carcinomas
NECs: Neuroendocrine carcinomas
RR: Risk ratio
Syn: Synaptophysin
